# Successful Conservative Management of Complicated *Brucella* Endocarditis

**DOI:** 10.14740/jmc5283

**Published:** 2026-03-04

**Authors:** Mashael M. Alhajri

**Affiliations:** Infectious Diseases Division, Department of Internal Medicine, Faculty of Medicine, Imam Abdulrahman Bin Faisal University, Dammam & King Fahad Hospital of the University, Al-Khobar, Saudi Arabia. Email: Mmalhajri7@gmail.com; mhajri@iau.edu.sa

**Keywords:** *Brucella* endocarditis, Conservative management, Paravalvular abscess, Prosthetic valve, Spondylitis, Stroke

## Abstract

*Brucella* endocarditis (BE) is a rare but life-threatening complication of brucellosis and remains the principal cause of disease-related mortality. Prosthetic-valve involvement is exceptionally uncommon and is usually managed with combined medical and surgical therapy because of the high risk of abscess formation and embolic complications. We report a 51-year-old man with a bioprosthetic aortic valve who presented with recurrent fever, weight loss, and acute neurological deficits following previously treated, culture-confirmed brucellosis related to consumption of unpasteurized goat milk. Diagnostic evaluation revealed an embolic ischemic stroke, prosthetic-valve vegetation complicated by a paravalvular aortic-root abscess, rising *Brucella melitensis* serological titers despite negative blood cultures, and concomitant brucellar spondylitis. A diagnosis of prosthetic-valve BE with systemic dissemination was established. Although urgent surgical intervention was recommended, the patient declined surgery and was treated conservatively with prolonged combination antimicrobial therapy. The outcome was favorable, with complete clinical recovery, significant neurological improvement, normalization of inflammatory markers, and complete echocardiographic resolution of vegetation and abscess while preserving ventricular function. This case supports the potential role of individualized conservative management in selected patients with complicated BE.

## Introduction

Brucellosis is a zoonotic infection caused by gram-negative, facultative intracellular bacteria of the genus *Brucella*. Despite global control efforts, the disease remains endemic in many regions worldwide, particularly the Middle East, the Mediterranean basin, South Asia, and parts of Africa and Latin America. In Saudi Arabia, brucellosis continues to represent a significant public health problem, primarily driven by the consumption of unpasteurized dairy products and close contact with infected livestock. While most patients present with a nonspecific systemic febrile illness, focal organ involvement may occur and is associated with increased morbidity and mortality [1, 2].

*Brucella* endocarditis (BE) is the most severe yet least frequent complication of brucellosis, occurring in approximately 1–2% of infected individuals. Despite its low incidence, BE accounts for more than 80% of brucellosis-related deaths [3]. The aortic valve is the most commonly affected site, followed by the mitral valve, whereas involvement of prosthetic valves is rare but particularly challenging. Prosthetic material, altered hemodynamics, and biofilm formation create a favorable environment for persistent infection, leading to progressive valvular destruction, paravalvular abscess formation, and embolic complications, often necessitating surgical intervention [4, 5].

BE diagnosis is frequently delayed because blood cultures are positive in fewer than half of cases, especially in patients who have received prior antibiotic therapy. As a result, diagnosis often depends on a combination of epidemiological exposure, serological testing, and echocardiographic evaluation. Transesophageal echocardiography (TEE) plays a pivotal role in detecting prosthetic-valve vegetations, paravalvular abscesses, and periannular extension, all of which are critical determinants of prognosis and management [6, 7].

Standard management of BE typically consists of prolonged combination antimicrobial therapy alongside surgical valve replacement, particularly in patients with heart failure, abscess formation, or embolic phenomena. However, surgical intervention may be contraindicated or declined by some patients. In such circumstances, conservative medical management becomes the only feasible therapeutic option. Nevertheless, the evidence supporting this approach remains limited and largely confined to isolated case reports and small case series [8].

In this report, we describe a complex case of prosthetic-valve BE complicated by paravalvular abscess, ischemic stroke, and spondylitis that was successfully managed with conservative antimicrobial therapy alone. This case highlights the importance of individualized treatment strategies and contributes to the limited body of literature supporting non-surgical management of complicated BE in carefully selected patients [9].

## Case Report

A 51-year-old Saudi male from Albaha, Saudi Arabia was admitted and managed at a tertiary care hospital in the Eastern Province of Saudi Arabia. He had a history of rheumatic heart disease diagnosed at 20 years of age and had undergone bioprosthetic aortic valve replacement 3 years before the current presentation. His past medical history also included type 2 diabetes mellitus, well-controlled on metformin, with no known immunodeficiency. He had no relevant family history of cardiac or infectious diseases. The patient reported frequent consumption of unpasteurized goat milk and occasional contact with livestock.

Five months before the current admission, he developed intermittent fever and lower back pain. Blood cultures at that time yielded *Brucella melitensis*, confirming brucellosis. He was treated orally with rifampicin 600 mg once daily and ciprofloxacin 500 mg twice daily for 6 weeks, resulting in clinical and laboratory improvement with complete resolution of symptoms.

Two months after completing therapy, the patient presented again with recurrent fever, malaise, anorexia, and unintentional weight loss of approximately 10 kg. A few days later, he developed a sudden onset of left-sided weakness affecting both the upper and lower limbs.

On examination, he was febrile and appeared fatigued but hemodynamically stable. Cardiovascular examination revealed a systolic murmur over the aortic area without signs of heart failure. The electrocardiogram showed first-degree atrioventricular block with a normal heart rate of 80/min. Neurological examination demonstrated left-sided hemiparesis with reduced muscle power and no other signs suggestive of spinal cord compression, such as loss of bowel or bladder control. No peripheral stigmata of infective endocarditis (IE) were observed. Abdominal examination was unremarkable with no organomegaly or tenderness. Table 1 provides a summary of clinical timeline events before and after the presentation of BE in the admitted case.

**Table 1:** Clinical Timeline of Events for the Admitted Case Before and After BE Presentation

Timeline	Clinical events and findings	Management	CRP (mg/L)	Procalcitonin (ng/mL)	Brucella serology titers	Outcome
5 months prior	Intermittent fever, lower back pain. Blood culture: *Brucella melitensis*	Rifampicin + ciprofloxacin for 6 weeks.	6	-	1:320	Symptom resolution
2 months post-treatment (admission)	Recurrent fever, malaise, 10 kg weight loss, followed by acute left-sided hemiparesis.	Hospital admission, diagnostic workup.	12	0.20	1:40 → 1:160	Embolic stroke confirmed on CT.
During inpatient phase (weeks 1–8)	Diagnosis of BE with paravalvular abscess and spondylitis. Patient declined surgery.	Initiated quadruple therapy: rifampicin, doxycycline, gentamicin, TMP-SMX. Inpatient monitoring.	14 → 10 → 3.37	0.01 → 0.03	1:320	Fever and back pain resolved. Neurological improvement. Echo: vegetation and abscess regressing.
Post-discharge (months 3–9)	Outpatient follow-up. Gentamicin was discontinued after the initial phase.	Continued oral therapy: rifampicin, doxycycline, TMP-SMX. Bi-weekly clinical and lab follow-up.	1.7	0.05	1:80	Clinically stable, afebrile. Independent in daily activities.
6-month follow-up	Asymptomatic. No signs of heart failure or infection relapse.	Completion of a prolonged antimicrobial course. Final echocardiogram.	Normal	Normal	1:40 (baseline)	Complete resolution: No vegetation/abscess on echo. Preserved valve function. No relapse.

BE: *Brucella* endocarditis; CRP: C-reactive protein; CT: computed tomography; Echo: echocardiography; TMP-SMX: trimethoprim-sulfamethoxazole.

### Investigations and diagnosis

Initial laboratory investigations demonstrated mildly elevated inflammatory markers, with a C-reactive protein (CRP) level of 12 mg/L and an erythrocyte sedimentation rate (ESR) of 17 mm/h, reflecting low-grade systemic inflammation. Complete blood count, renal function tests, liver enzymes, urine analysis, and electrolyte levels were within normal limits, with no evidence of cytopenia, renal impairment, or hepatic dysfunction. These relatively modest laboratory abnormalities were consistent with the indolent nature of *Brucella* infection and its tendency to present with subtle inflammatory responses, particularly in patients previously exposed to antimicrobial therapy.

**Figure 1:**
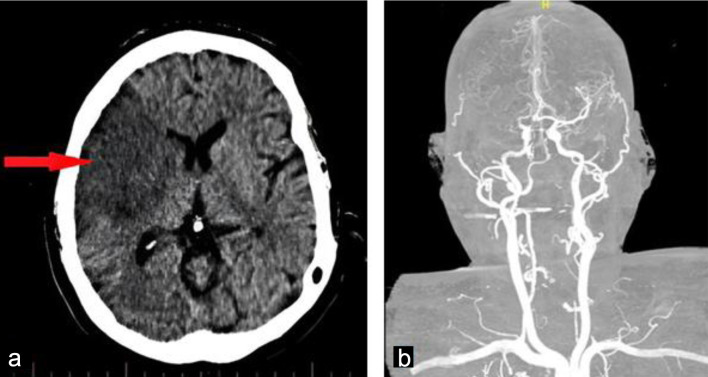
Admission brain computed tomography without contrast obtained on admission showing a well-defined hypodense lesion in the right (arrow), consistent with an acute embolic ischemic infarction (a). Cerebral angiography showed occlusion of the right middle cerebral artery (b).

Neuroimaging was promptly performed following the onset of acute focal neurological deficits. Non-contrast computed tomography (CT) of the brain revealed a well-demarcated infarction within the right middle cerebral artery territory, consistent with an embolic ischemic stroke (Fig. 1). This finding raised immediate concern for a cardioembolic source in the context of the patient’s prosthetic aortic valve and recurrent systemic symptoms.

**Figure 2:**
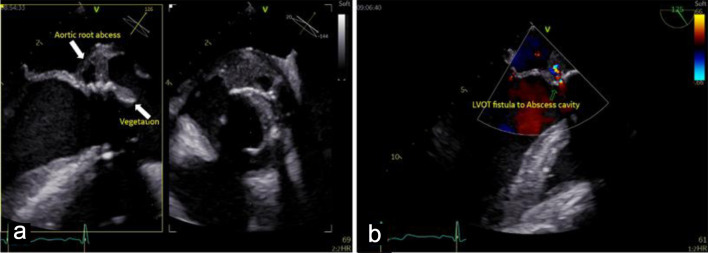
Mid-esophageal view transesophageal echocardiogram (TEE) demonstrates: (a) pedunculated vegetation (lower arrow, measured 3 × 6 mm) on the bioprosthetic aortic valve cusp, and paravalvular aortic-root abscess (upper arrow), indicating advanced infective endocarditis. (b) Doppler echocardiogram shows left ventricular outflow tract (LVOT) fistula to the abscess cavity.

**Figure 3:**
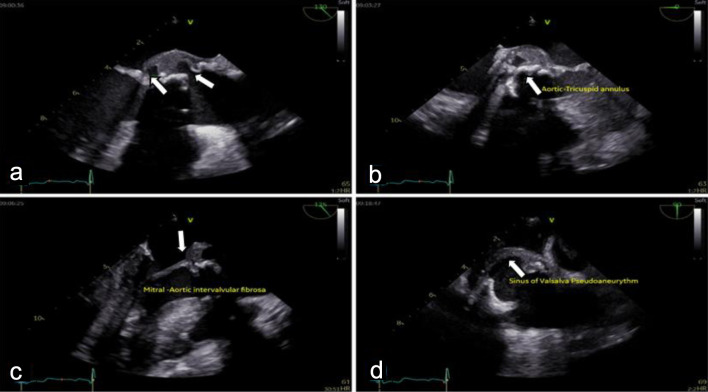
Transesophageal echocardiography (TEE) shows destructive prosthetic aortic valve endocarditis with peri-annular extension: (a) large mobile vegetation attached to the bioprosthetic aortic valve cusps, (b) extension of infection to the aortic annulus with paravalvular aortic root abscess formation, (c) destruction of the mitral–aortic intervalvular fibrosa (MAIVF) (arrow), and (d) a sinus of Valsalva pseudoaneurysm (arrow).

Cardiac evaluation was initiated with transthoracic echocardiography (TTE), which identified a mobile, pedunculated vegetation measuring approximately 3 × 6 mm attached to the prosthetic aortic valve suture line. The vegetation was associated with mild aortic regurgitation, while overall prosthetic valve function remained preserved. Left ventricular systolic function was maintained, with an estimated ejection fraction of 54%, and no signs of heart failure or significant ventricular dilation were observed. To further characterize the extent of valvular and periannular involvement, TEE was subsequently performed. TEE confirmed the presence of the prosthetic-valve vegetation and revealed a paravalvular aortic-root abscess (Fig. 2) extending into the sinuses of Valsalva and involving the mitral–aortic intervalvular fibrosa. These findings were indicative of advanced prosthetic-valve IE with periannular extension (Fig. 3), a condition traditionally associated with poor prognosis and strong indications for urgent surgical intervention.

Microbiological evaluation included repeated blood cultures, all of which remained negative, likely due to prior antibiotic exposure during earlier treatment for brucellosis. In view of this, serological testing played a pivotal diagnostic role. *Brucella melitensis* serology (enzyme-linked immunosorbent assay (ELISA) method) demonstrated a significant rise in antibody titers from 1:40 to 1:320, with concomitant elevation of both IgM and IgG levels, strongly supporting relapsed brucellosis with active infection. These findings, in conjunction with the patient’s epidemiological exposure history and compatible clinical presentation, reinforced the diagnosis despite culture negativity.

Given the patient’s complaint of persistent lower back pain, further evaluation for extra-cardiac involvement was undertaken. Magnetic resonance imaging (MRI) of the lumbar spine demonstrated vertebral body signal changes and inflammatory features consistent with brucellar spondylitis, confirming systemic dissemination of the infection. Abdomen ultrasound (US) was unremarkable with no organomegaly or abscess formation, nor in the liver, spleen, or pancreas.

Integrating clinical presentation, epidemiological risk factors, serological evidence, and multimodal imaging findings, the final diagnosis was established as prosthetic-valve BE complicated by paravalvular aortic-root abscess formation, embolic ischemic stroke, and brucellar spondylitis.

### Treatment and outcomes

Following confirmation of prosthetic-valve BE complicated by a paravalvular aortic-root abscess and embolic ischemic stroke, the patient was urgently evaluated by the cardiothoracic surgery team. Given the presence of periannular extension and systemic embolization, early surgical valve replacement was strongly recommended in accordance with international IE guidelines. However, after extensive multidisciplinary discussions outlining the risks, benefits, and potential consequences of surgical and non-surgical approaches, the patient firmly declined operative intervention.

In view of this decision, a conservative medical strategy was adopted with close inpatient monitoring. The patient was initiated on an intensive quadruple antimicrobial regimen comprising rifampicin 900 mg orally once daily, doxycycline 100 mg orally twice daily, gentamicin 300 mg intravenously once daily, and trimethoprim–sulfamethoxazole (two double-strength tablets twice daily).

The patient remained hospitalized for nearly 2 months under continuous multidisciplinary supervision involving infectious disease specialists, cardiologists, neurologists, physiotherapists, and clinical pharmacists. During this period, he demonstrated gradual but sustained clinical improvement, with complete resolution of fever, back pain, and constitutional symptoms. Serial laboratory assessments showed progressive normalization of inflammatory markers, including C-reactive protein (CRP) and erythrocyte sedimentation rate (ESR). Repeated TTE and TEE revealed a gradual reduction in vegetation size and marked regression of the paravalvular abscess without deterioration of prosthetic-valve function or development of heart failure.

After completion of the inpatient phase, gentamicin was discontinued to minimize the risk of nephrotoxicity, and the patient was transitioned to prolonged oral therapy with rifampicin, doxycycline, and trimethoprim–sulfamethoxazole for an additional 6 months. He was closely followed in the outpatient setting at bi-weekly intervals. Adherence to antimicrobial therapy was verified through structured clinical interviews, pharmacy refill documentation, and serial laboratory monitoring, with no significant adverse drug reactions or treatment-limiting toxicities observed.

Neurological recovery was supported by early initiation and continuation of structured physiotherapy and rehabilitation. The patient demonstrated progressive improvement in motor strength, reaching grade 4 out of 5 on the Medical Research Council scale, with restoration of functional independence and the ability to perform activities of daily living without assistance. At 6-month follow-up, he remained afebrile and clinically stable, with no evidence of recurrent infection.

Serial follow-up echocardiography confirmed preserved prosthetic aortic-valve function, complete resolution of the previously documented vegetation, and absence of residual or recurrent paravalvular abscess. No relapse of brucellosis or extra-cardiac involvement was detected during follow-up.

## Discussion

This case, highlighting the successful outcome of prosthetic-valve BE complicated by paravalvular abscess, embolic strike, and spondylitis, with conservative medical interventions solely, presents a compelling instance as a notable exception to standard BE management paradigms. The prognosis and clinical course of IE caused by *Brucella* species differ meaningfully from those caused by more common bacterial or fungal pathogens. BE typically presents with a more subacute and destructive course, carries a significant mortality risk reported at 11.8% even with treatment, and is an independent risk factor for perivalvular complications compared to non-*Brucella* IE [10, 11]. Furthermore, specific prognostic markers are unique to *Brucella* IE, such as a high baseline Wright Standard Tube Agglutination (STA) titer (≥ 1:1,280), which independently predicts worse outcomes [5]. This distinct profile underscores that *Brucella* is not merely a rare cause of IE but one that necessitates specific diagnostic suspicion and tailored management strategies.

These conservative clinical outcomes are rare, as shown in Supplementary Material 1 (jmc.elmerpub.com), where only seven cases documented the successful conservative management of high-risk prosthetic valve BE [6, 12–15]. For patients presenting with complicated IE, established clinical guidelines [16] and recommendations emphasize the need for close multidisciplinary monitoring [17–19]. This surveillance is anchored on serial echocardiography, especially TEE, to monitor vegetation size, valvular function, and the resolution of complications such as abscesses. Concurrently, tracking inflammatory markers (CRP, ESR) and Brucella serological titers provides essential laboratory correlates of treatment response and helps guide therapy duration, with a significant and sustained decrease in titers often indicating successful microbial eradication [5, 14, 19]. Furthermore, vigilant monitoring for embolic phenomena through the clinical assessment and drug-related toxicities, such as nephrotoxicity, is paramount throughout the treatment course [18].

The clinical events in this case offer insights into the potential of non-surgical strategies even in the presence of abscess formation in selected high-risk patients.

Several unique factors highlight the distinctive nature of the BE presentation in this particular case. Firstly, the rare involvement of a bioprosthetic aortic valve, which has been significantly associated with grave prognosis, is attributed to the high rates of perivalvular extensions and embolic complications [20–22]. Secondly, brucella infection relapse after a standard course of antimicrobial therapy underscores the significantly documented challenge of eradicating *Brucella* biofilm on prosthetic material as articulated by Moohialdin et al [23] and Mikzinski et al [24]. A third distinctive feature of this case was the presentation with concurrent life-threatening complications, including a paravalvular aortic-root abscess and an embolic ischemic stroke, along with the systemic dissemination evidenced by brucellar spondylitis. Interestingly, despite these high-risk features and a strong recommendation for urgent surgery, the patient’s refusal of surgery resulted in a purely medical approach, which contributed to complete clinical and echocardiographic resolution. Consequently, this favorable outcome adds a significant data point to the sparse literature on non-surgical management of complicated prosthetic-valve BE [6, 12–15].

According to the European Society of Cardiology (ESC), patients presenting with high-risk, class I prosthetic valve endocarditis complicated by perivalvular extension, heart failure, or recurrent embolic events warrant early intervention [25–27]. Studies in this regard present significant evidence underscoring reduced mortality rates ranging from 10% and 26%, with long-term survival rates documented at 70% after 5 years and 60% after 10 years for in-hospital survivors following surgical interventions in high-risk scenarios such as the one presented in this paper [28–30].

For BE specifically, expert opinions and systematic reviews advocate for a combined-surgical strategy, especially in the presence of prosthetic material, large vegetation (> 10 mm), abscess formation, or embolic phenomena [21, 31]. Several factors likely contributed to the favorable outcome in this patient, including preserved left ventricular function, absence of heart failure, relatively small vegetation size, and hemodynamic stability. In addition, early initiation of a synergistic multidrug regimen composed of a quadruple combination of rifampicin, doxycycline, gentamicin, and trimethoprim-sulfamethoxazole (TMP-SMX) and close echocardiographic and clinical monitoring were critical. The slow replication rate of *Brucella* species may allow a window for infection control before irreversible structural damage occurs [5]. In this case, initial therapy with intravenous gentamicin provided rapid bactericidal activity. The rapid bacterial activity was followed by a prolonged oral antimicrobial phase aimed at complete eradication and prevention of relapse, as supported by previous studies [32, 33]. Notably, the initial intensive inpatient phase with intravenous gentamicin likely provided rapid bacterial reduction while the prolonged oral phase aimed for complete eradication and relapse prevention [5, 34].

Evidence supporting the conservative management for BE, especially in complex cases, to date remains significantly scarce owing to the high mortality rates and risks of relapses associated with conservative management [5]. Cohen et al document a case report of complete recovery in a 55-year-old man treated with doxycycline at 200 mg/day, rifampicin at 900 mg/day, and parenteral gentamicin at 240 mg/day, completely resolving the diastolic murmur and decreasing the valvular vegetation. After 22 months post-hospitalization, the patient’s condition remained completely satisfactory [35]. In turn, Fonseca et al report on the complete resolution of prosthetic valve BE in a 60-year-old female farmer with antibiotic interventions, including doxycycline 100 mg twice a day and oral rifampicin 600 mg once daily solely [6].

Similarly, Karaoglan et al document three cases of a 42-year-old woman, a 27-year-old male sheep herder, and a 56-year-old male patient successfully treated for prosthetic valve BE following conservative treatments, primarily antibiotics such as combined doxycycline and rifampicin or doxycycline, rifampicin, and trimethoprim-sulfamethoxazole for between 6 weeks and 4 months following BE diagnosis [12]. A similar outcome was also reported by Murdaca et al, Mert et al, and Lee et al, in patients presenting with prosthetic mitral valve BE with a combination of antimicrobial therapy [13–15]. To the best of our knowledge, this is the second case worldwide reported to have successful conservative treatment in patients with prosthetic valve BE complicated by abscess formation.

### Conclusion

This case illustrates that, although surgical intervention remains the standard of care for prosthetic-valve BE complicated by abscess formation and embolic events, carefully selected patients who refuse or are not candidates for surgery may achieve favorable outcomes with prolonged, closely monitored conservative antimicrobial therapy. Early diagnosis, preserved cardiac function, use of a synergistic multidrug regimen, and rigorous multidisciplinary follow-up were critical to treatment success. This report contributes to the limited evidence supporting individualized non-surgical management strategies for complicated BE. Larger prospective studies are needed to define selection criteria and establish evidence-based guidelines for conservative management of BE.

## Supplementary Material

Suppl 1 Summary of reported cases of prosthetic valve *Brucella* endocarditis managed conservatively.

## Data Availability

All data generated or analyzed during this study are included in this published article. Additional information is available from the corresponding author upon reasonable request, in accordance with patient confidentiality and ethical considerations.

## References

[1] Atluri VL, Xavier MN, de Jong MF, den Hartigh AB, Tsolis RM (2011). Interactions of the human pathogenic Brucella species with their hosts. Annu Rev Microbiol.

[2] Bhat SA, Guroo FA, Koul AN, Mantoo S, Siraj F, Soharwardy MY (2024). Brucella endocarditis: a case series. Cureus.

[3] Jin M, Fan Z, Gao R, Li X, Gao Z, Wang Z (2023). Research progress on complications of Brucellosis. Front Cell Infect Microbiol.

[4] Ferreira P, Gama P, Correia J, Nunes L, Pipa J, Nascimento C, Alexandre JC (2008). Brucella endocarditis—case report and literature review. Rev Port Cardiol.

[5] Basaran S, Simsek-Yavuz S, Saricaoglu ME, Aydin M, Aygun G, Azap A, Azap O (2025). A systematic review and analysis of Brucella endocarditis cases. Anatol J Cardiol.

[6] Fonseca JP, Pereiro T, Dos Santos DP, Correia JM, Capelo J, Carragoso A (2018). Successful Management of Prosthetic Valve Brucella Endocarditis with Antibiotherapy Alone. Eur J Case Rep Intern Med.

[7] Habib G, Lancellotti P, Antunes MJ, Bongiorni MG, Casalta JP, Del Zotti F, Dulgheru R (2015). 2015 ESC Guidelines for the management of infective endocarditis: the task force for the management of infective endocarditis of the European Society of Cardiology (ESC). Endorsed by: European Association for Cardio-Thoracic Surgery (EACTS), the European Association of Nuclear Medicine (EANM). Eur Heart J.

[8] Koruk ST, Erdem H, Koruk I, Erbay A, Tezer-Tekce Y, Erbay AR, Dayan S (2012). Management of Brucella endocarditis: results of the Gulhane study. Int J Antimicrob Agents.

[9] Alici H, Ercan S, Davutoglu V (2014). Brucella infective endocarditis. Cor Vasa.

[10] Alfakeeh S, Alghanem RF, Bin Obaid S, Alsuwayhib A, Al Kawabah G, Abanamy R, Bosaeed M (2024). Clinical characteristics and outcome of brucella endocarditis: a case series. Infect Drug Resist.

[11] Pan S, Zhao Y, Zhou K, Chen S, Maimaitiming M, Wu J, Tuerxun M (2024). Incidence and outcomes of brucella endocarditis in a high-prevalence area: a single-center study. J Epidemiol Glob Health.

[12] Karaoglan I, Namiduru M, Baydar I, Zer Y, Erdem M, Kuvandik C (2009). Three cases of *brucella* prosthetic valve endocarditis cured with medical treatment. ANKEM Derg.

[13] Murdaca G, Colombo BM, Caiti M, Cagnati P, Massa G, Puppo F (2007). Remission of brucella endocarditis in a patient with mitral valve mechanical prosthesis by antibiotic therapy alone: a case report. Int J Cardiol.

[14] Mert A, Kocak F, Ozaras R, Tabak F, Bilir M, Kucukuglu S, Ozturk R (2002). The role of antibiotic treatment alone for the management of Brucella endocarditis in adults: a case report and literature review. Ann Thorac Cardiovasc Surg.

[15] Lee SA, Kim KH, Shin HS, Lee HS, Choi HM, Kim HK (2014). Successful medical treatment of prosthetic mitral valve endocarditis caused by brucella abortus. Korean Circ J.

[16] Delgado V, Ajmone Marsan N, de Waha S, Bonaros N, Brida M, Burri H, Caselli S (2023). 2023 ESC Guidelines for the management of endocarditis. Eur Heart J.

[17] Lau L, Baddour L, Fernandez Hidalgo N, Brothers TD, Kong WKF, Borger MA, Duval X (2025). Infective endocarditis: it takes a team. Eur Heart J.

[18] Rajani R, Klein JL (2020). Infective endocarditis: A contemporary update. Clin Med (Lond).

[19] Roy AS, Hagh-Doust H, Abdul Azim A, Caceres J, Denholm JT, Dong MQD, King M (2023). Multidisciplinary Teams for the Management of Infective Endocarditis: A Systematic Review and Meta-analysis. Open Forum Infect Dis.

[20] Alassiri AK, Alshair FM, Fatani MA, Baghaffar AH (2023). Aortic root abscess and Brucella endocarditis in a patient with mechanical aortic valve prosthesis: a case report. J Surg Case Rep.

[21] Taamallah K, Hammami F, Gharsallah H, Koubaa M, Ben Jemaa M, Fehri W (2021). Brucella prosthetic valve endocarditis: a systematic review. J Saudi Heart Assoc.

[22] Al Dahouk S, Schneider T, Jansen A, Nockler K, Tomaso H, Hagen RM, Scholz HC (2006). Brucella endocarditis in prosthetic valves. Can J Cardiol.

[23] Moohialdin NN, Shamsodini A, Wilson SK, Abdeljaleel O, Alnadhari I, Abdulmuhsin AS (2020). First reported case of penile prosthesis infection from brucellosis: case report. Afr J Urol.

[24] Mikzinski P, Kraus K, Widelski J, Paluch E (2024). Modern microbiological methods to detect biofilm formation in orthopedy and suggestions for antibiotic therapy, with particular emphasis on Prosthetic Joint Infection (PJI). Microorganisms.

[25] Peijster AJL, van Nieuwkoop C, Keunen RWM, Felix S, van Welzen BJ, Kouijzer IJE, Boel CHE (2026). 2023 European Society of Cardiology guidelines for the management of infective endocarditis : Statement of endorsement by the NVVC Full version. Neth Heart J.

[26] Brugiatelli L, Patani F, Lofiego C, Benedetti M, Capodaglio I, Giulia P, Matteo F (2025). Multimodality imaging in infective endocarditis: a clinical approach to diagnosis. Medicina (Kaunas).

[27] Yanagawa B, Pettersson GB, Habib G, Ruel M, Saposnik G, Latter DA, Verma S (2016). Surgical management of infective endocarditis complicated by embolic stroke: practical recommendations for clinicians. Circulation.

[28] Gopal K, Radhakrishnan RM, Jose R, Krishna N, Varma PK (2024). Outcomes after surgery for infective endocarditis. Indian J Thorac Cardiovasc Surg.

[29] Varela Barca L, Lopez-Menendez J, Navas Elorza E, Moya Mur JL, Centella Hernendez T, Redondo Palacios A, Fajardo ER (2019). Long-term prognosis after surgery for infective endocarditis: Distinction between predictors of early and late survival. Enferm Infecc Microbiol Clin (Engl Ed).

[30] Mokhles MM, Ciampichetti I, Head SJ, Takkenberg JJ, Bogers AJ (2011). Survival of surgically treated infective endocarditis: a comparison with the general Dutch population. Ann Thorac Surg.

[31] Sidik AI, Khavandeev ML, Dontsov VV, Esion GA, Hossain ML, Njoya Mbombo EJ, Karpenko I (2025). Surgical management of infective endocarditis: indications, techniques, and outcomes. Cureus.

[32] Guimaraes ES, Gomes MTR, de Araujo A, Ramos KKS, Oliveira SC (2025). Pathogenicity and virulence of brucella: strategies for metabolic adaptation and immune evasion. Virulence.

[33] Jiao H, Zhou Z, Li B, Xiao Y, Li M, Zeng H, Guo X (2021). The mechanism of facultative intracellular parasitism of brucella. Int J Mol Sci.

[34] Chaves BJ, Tadi P (2025). Gentamicin. StatPearls.

[35] Cohen N, Golik A, Alon I, Zaidenstein R, Dishi V, Karpuch J, Zyssman I (1997). Conservative treatment for Brucella endocarditis. Clin Cardiol.

