# Supplementary material for: Successful Conservative Management of Complicated Brucella Endocarditis
Source: J Med Cases. 2026 Mar 4;17(4):176–82. doi: 10.14740/jmc5283 (PMC12978408; doi:10.14740/jmc5283)
Supplement: Suppl 1 — Summary of reported cases of prosthetic valve Brucella endocarditis managed conservatively. [file jmc-17-04-176-s001.docx]

**Suppl 1.** Summary of Reported Cases of Prosthetic Valve Brucella Endocarditis Managed Conservatively

| **Author (year)** | **Age/sex** | **Prosthetic valve type** | **Complications** | **Antimicrobial regimen** | **Duration of therapy** | **Outcome** |
| --- | --- | --- | --- | --- | --- | --- |
| Present Case (2025) | 51/M | Bioprosthetic aortic | Paravalvular abscess, embolic stroke, spondylitis | Rifampicin, doxycycline, gentamicin, TMP-SMX (IV then oral) | 6 months | Complete clinical & echocardiographic resolution |
| Fonseca et al. (2018) [6] | 60/F | Mechanical aortic | Vegetations, spondylitis. No abscess, no heart failure | Doxycycline, rifampicin, and IV gentamicin combination | 9 months | Complete resolution |
| Karaoğlan et al. (Case 1) [18] | 42/F | Mechanical mitral | Vegetation (0.8x0.9 cm) | Rifampicin, doxycycline, TMP-SMX (after initial aminoglycoside) | 12 months | Resolved with sequelae: CNS microembolization during treatment, motor deficit. |
| Karaoğlan et al. (Case 2) [18] | 27/M | Mechanical aortic | Vegetation (1.4x0.6 cm) | Rifampicin, doxycycline, ceftriaxone (initial) | 6 months | Complete resolution |
| Karaoğlan et al. (Case 3) [18] | 56/M | Mechanical aortic | Periannular abscess (0.3x0.5 cm) | Rifampicin, doxycycline | 6 months | Complete resolution (abscess resolved in 1 month) |
| Murdaca et al. (2007) (1) | 63/F | Mechanical mitral | Vegetation. No abscess, no ~~HF~~ heart failure | Doxycycline, rifampicin, streptomycin (initial) | 6 weeks | Complete resolution |
| Mert et al. (2002) [14] | 24/M | Mechanical aortic | Heart failure, emboli (no abscess) | Doxycycline, rifampicin, streptomycin (initial) | 6 months | Complete resolution |
| Lee et al. (2014) [15] | 60/F | Mechanical mitral | None reported | Doxycycline, rifampicin, TMP-SMX (initial) | 6 months | Complete resolution |
